# The Role of MicroRNA in the Pathogenesis of Acute Kidney Injury

**DOI:** 10.3390/cells13181559

**Published:** 2024-09-16

**Authors:** Estera Bakinowska, Kajetan Kiełbowski, Andrzej Pawlik

**Affiliations:** Department of Physiology, Pomeranian Medical University, 70-111 Szczecin, Poland; esterabakinowska@gmail.com (E.B.); kajetan.kielbowski@onet.pl (K.K.)

**Keywords:** acute kidney injury, non-coding RNA, microRNA

## Abstract

Acute kidney injury (AKI) describes a condition associated with elevated serum creatinine levels and decreased glomerular filtration rate. AKI can develop as a result of sepsis, the nephrotoxic properties of several drugs, and ischemia/reperfusion injury. Renal damage can be associated with metabolic acidosis, fluid overload, and ionic disorders. As the molecular background of the pathogenesis of AKI is insufficiently understood, more studies are needed to identify the key signaling pathways and molecules involved in the progression of AKI. Consequently, future treatment methods may be able to restore organ function more rapidly and prevent progression to chronic kidney disease. MicroRNAs (miRNAs) are small molecules that belong to the non-coding RNA family. Recently, numerous studies have demonstrated the altered expression profile of miRNAs in various diseases, including inflammatory and neoplastic conditions. As miRNAs are major regulators of gene expression, their dysregulation is associated with impaired homeostasis and cellular behavior. The aim of this article is to discuss current evidence on the involvement of miRNAs in the pathogenesis of AKI.

## 1. Introduction

Acute kidney injury (AKI) is a term that represents several disorders that are associated with increased serum levels of creatinine and decreased glomerular filtration rates (GFR) [1]. The prevalence of AKI is estimated at approximately 20%, but it can be higher in critically ill patients hospitalized in intensive care units (ICUs) [2,3]. Renal impairment in AKI may lead to fluid overload, ionic disorders, and metabolic acidosis. Depending on the extent of these disorders, patients may need to undergo a renal replacement therapy (RRT) such as hemofiltration and hemodialysis, among other methods [4]. As in every pathological condition, understanding the pathogenetic mechanisms and pathophysiology is important to improve the prevention, diagnosis, and treatment of the disease. Furthermore, the identification of novel therapeutic targets and signaling pathways implicated in the pathogenesis of particular diseases could prevent the development of subsequent complications. In recent years, non-coding RNAs (ncRNAs) have been widely investigated as molecules that are frequently dysregulated in numerous conditions [5,6], including renal diseases [7]. ncRNAs are important regulators of gene expression, and thus they significantly affect cellular behavior. For instance, miR-19 mediates aldosterone responsiveness and controls sodium transport [8]. Several classes of RNA molecules belong to the ncRNA family, including microRNA (miRNA), long non-coding RNA (lncRNA), and circular RNA (circRNA), among others. The aim of this review is to summarize the current evidence relating to the involvement of miRNAs in the pathogenesis of AKI.

## 2. The Role of MicroRNA in the Pathogenesis of Acute Kidney Injury—In Vitro and Animal Models

### 2.1. Brief Overview of miRNA Biogenesis and Functionality

miRNAs were discovered in *Caenorhabditis elegans* in 1993; to this day, 2654 human miRNAs have been described [9,10]. The molecules are responsible for cell homeostasis by controlling gene expression. Their biogenesis begins when chromosomal DNA undergoes transcription from RNA polymerase II into primary miRNA transcripts (pri-miRNA) which later become stem-loop like structures which are precursor miRNAs (pre-miRNAs) with approximately 70 nucleotides by RNase III Drosha [11,12]. Subsequently, pre-miRNA is transported with specific carrier exportin-5 from the nucleus to the cytoplasm [13,14]. In the cytoplasm, pre-miRNAs are separated by Dicer/TRBP enzyme complexes into single-stranded RNA miRNA duplexes, while one strand is degraded [15]. miRNAs duplexes are 21–24 nucleotides in size [16,17]. Subsequently, the guide strand interacts with the Argonaute (AGO) protein of an RNA-induced silencing complex (RISC). The complex interferes with complimentary mRNA to induce its destabilization or translational repression [18]. Thus, the classical mechanism of action of miRNA molecules involves negative post-transcriptional regulation [19,20]. These molecules are located both intra- and extra-cellularly, making it possible to monitor their expression in blood samples [21]. Moreover, they have been isolated from urine, follicular fluid, cerebrospinal fluid (CSF), and saliva [22].

### 2.2. miRNA in Sepsis-Induced AKI

The classical function of miRNAs in regulating gene expression is to suppress translation after binding to their target mRNA. Some studies have also demonstrated that miRNAs can enhance gene expression [23]. Under pathological conditions, the expression of miRNAs changes in the affected tissue, as well as in biological fluids. Diseased tissues contribute to the altered expression profile of circulating molecules by secreting extracellular vesicles that contain miRNAs. In addition, as a result of tissue damage and cellular death, miRNAs are released into the extracellular space. In the case of renal tissue, different patterns of expression of RNA molecules can be detected in urine.

Several studies have demonstrated that the state of AKI significantly alters the expression of miRNAs. In a mouse model of hypoxia-induced renal damage, 18 miRNAs were found to be differentially expressed [24]. Moreover, poisoning induced alterations in urinary expression of miRNA molecules [25]. Such studies demonstrate that pathological conditions are associated with altered expression of miRNAs. Importantly, since these agents are significant gene expression regulators, their dysregulated presence can mediate the progression of AKI. 

**Figure 1:**
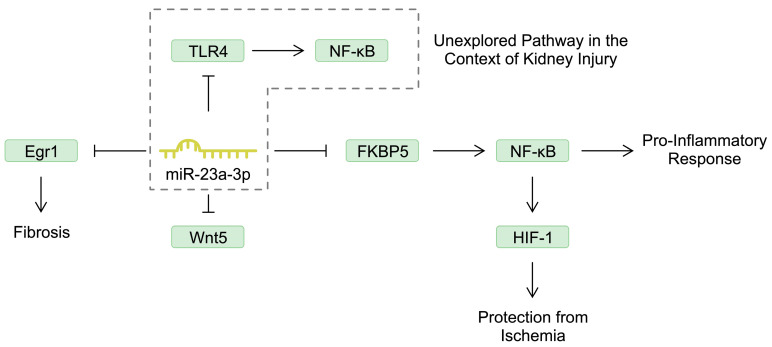
The involvement of miR-23a-3p in several pathways that could regulate the progression of acute kidney injury.

Renal damage during AKI development is associated with inflammatory responses and the increased expression and release of pro-inflammatory cytokines, chemokines, and adhesion molecules, as well as with the infiltration of immune cells [26]. miRNAs are known to regulate the expression of genes encoding inflammatory molecules. Nuclear factor kappa beta (NF-κB) is a major regulator of inflammatory responses that is implicated in the pathogenesis of several diseases [27,28]. Reducing the activity of NF-κB has also been suggested to induce positive effects in septic AKI [29]. miR-23a-3p is one of the molecules that is downregulated in patients with septic AKI [30]. An in vitro study demonstrated that stimulating HK-2 cells (proximal tubule epithelial cells) with lipopolysaccharide (LPS) reduced the expression of miR-23a-3p. Mechanistically, this miRNA negatively regulated FKBP prolyl isomerase 5 (FKBP5), which was associated with suppressed apoptosis and the release of inflammatory mediators (IL-8, IL-6). By targeting FKBP5, miR-23a-3p inactivates NF-κB, thus suppressing inflammatory responses [31]. Interestingly, NF-κB not only enhances inflammation, but can also induce protective mechanisms for the kidney. Specifically, it can stimulate the expression of hypoxia-inducible factor-1 (HIF-1), which can protect against ischemic conditions [32]. It is widely known that miRNAs are involved in a broad network of interactions and frequently target numerous genes. In the case of miR-23a-3p, Ye et al. [33] found that the molecule also negatively regulates Wnt5a, an element of the Wnt/β-catenin signaling pathway. The Wnt/β-catenin pathway plays an important role in kidney development and is considered to be protective in kidney injury. However, some evidence also suggests that this pathway could mediate the progression of AKI towards chronic kidney disease (CKD), possibly by modulating fibrosis [34]. In the previously mentioned study, Ye and colleagues [33] demonstrated that miR-23a-3p could mitigate kidney damage by downregulating Wnt5. In another study, miR-23a-3p suppressed the expression of pro-inflammatory mediators in HK-2 cells by targeting early growth response 1 (Egr1), a molecule suggested to be involved in the process of fibrosis [35]. miR-23a-3p might also play a role in the progression of AKI to CKD. Moreover, miR-23a-3p can also target toll-like receptor 4 (TLR4), as demonstrated in other cellular models [36,37]. Stimulating TLR4 can induce an intracellular signaling pathway that is myeloid differentiation primary response gene 88 (MyD88)-dependent and subsequently activates NF-κB, contributing to the pro-inflammatory response [38]. Stimulation of this pathway aggravates kidney cell injury [39]. A detailed review of the associations between TLR4 and AKI was conducted by Vázquez-Carballo et al. [40]. These studies demonstrate the broad interactions of miR-23a-3p that could take part in various mechanisms associated with AKI or with progression to CKD (Figure 1). Future studies should examine the miR-23a-3p/TLR4 axis in the model of AKI. 

miR-106a is another miRNA involved in the pathogenesis of renal diseases [41]. In contrast to miR-23a-3p, miR-106a is upregulated in septic patients. Stimulation of mouse kidney epithelial cells with LPS decreases their viability, which could be restored by transfection of miR-106 inhibitor. Similarly, inhibiting the molecule could reduce LPS-induced expression of pro-inflammatory mediators, such as IL-6, TNF-α, and IL-1β. Mechanistically, miR-106a could target and negatively regulate the expression of thrombospondin 2, a protein with antiangiogenic effects [42]. However, in HK-2 cells, miR-106a-5p was found to target high-mobility group 1 (HMGB1), an alarmin that stimulates inflammatory responses. In AKI mouse models, the expression of HMGB1 is elevated and associated with podocyte injury [43,44]. The signaling of HMGB1 involves stimulation of the TLR4/NF-κB pathway. miR-22 suppresses septic AKI by targeting the HMGB1/TLR4/NF-κB signaling pathway [45].

IL-6 is a pleiotropic cytokine that is classically associated with activation of the JAK/STAT signaling pathway. It is considered to exert both pro- and anti-inflammatory effects, but in inflammatory and malignant diseases it usually induces inflammatory reactions. Conflicting results have been published regarding the role of IL-6 in the pathogenesis of AKI. The expression of IL-6 increases in animal AKI models, and cytokine deficiency could prevent the development of renal injury. Furthermore, knockdown of IL-6 suppressed infiltration with neutrophils, an important cellular population that plays a role in AKI progression [46]. Furthermore, in pediatric patients undergoing cardiac surgery, elevated levels of IL-6 were observed in patients that developed post-operative AKI [47]. In patients admitted to the ICU, higher concentrations of IL-6 were associated with lower urine outputs within 72 h and a greater risk of persistent anuria. In contrast, this cohort demonstrated a lower risk of persistent AKI development [48]. However, in mice injected with cisplatin, knockout of IL-6 was associated with increased acute renal injury [49]. Moreover, IL-6 trans-signaling has been suggested to induce beneficial effects by stimulating anti-oxidating and repair mechanisms [50]. Thus, the role of IL-6 seems to be complex and might depend on the cellular context and the type of underlying injury. The IL-6 pathway is also regulated by miRNAs. Specifically, in a septic mouse model of AKI, the expression of miR-26a-5p is induced. This molecule has been suggested to stimulate protective mechanisms by targeting and downregulating IL-6 expression. Interestingly, the induction of miR-26a-5p expression was found to depend on NF-κB, further confirming the involvement of this transcription factor in protective mechanisms in the kidney [51]. Similarly to other RNA molecules, miR-26a-5p is implicated in a wider interaction network. Li et al. demonstrated that HK-2 cells can secrete this miRNA in exosomes. Blocking the release of exosomes could increase the expression of mir-26a-5p in proximal tubule cells. Consequently, increased expression of the molecule could inhibit the inflammatory response induced by bovine serum albumin. Specifically, miR-26a-5p could reduce protein expression of IL-6. Mechanistically, miR-26a-5p was found to target CHAC1, a molecule that enhances the activity of NF-κB and the expression of pro-inflammatory mediators. Therefore, these studies might demonstrate how different pro-inflammatory environments might change the mechanisms induced by NF-κB signaling [52]. In unilateral ureteral occlusion (UUO) mouse models, miR-26a-5p was also found to suppress fibrotic mechanisms [53]. Modulating the expression of miR-26a-5p could be an interesting approach for future studies, as its anti-inflammatory properties have been confirmed in other disease models as well [54,55,56]. 

Under septic conditions that occur in the development of AKI, other signaling pathways play an important role as well. Specifically, phosphatase and tensin homolog (PTEN) has been suggested to regulate renal function. This molecule is a member of several pathways, but one of the most frequently investigated signaling pathways associated with PTEN is the PI3K/AKT/mTOR pathway, which regulates major cellular functions. In a sepsis rat model, elevated expression of PTEN was accompanied by the decreased expression of miR-22-3p. Importantly, the induction of miR-22-3p in an in vivo experiment resulted in suppressed apoptosis of kidney tissue, inflammatory response, and markers of renal injury. Similarly, miR-22-3p reduced septic damage in HK-2 cells by targeting PTEN [57]. Another miRNA that regulates the expression of PTEN and the progression of AKI is miR-93. Zhan and colleagues demonstrated that its expression was reduced in kidney tissue obtained from septic AKI mouse models. In an in vitro experiment, miR-93 enhanced the viability of LPS-pretreated renal cells and reduced the expression of inflammatory mediators (IL-6, TNF-α, and IL-1β). By targeting PTEN, miR-93 stimulated the activity of the AKT/mTOR signaling pathway [58]. Similar results were observed regarding the miR-214 molecule [59]. However, one of the most investigated miRNAs targeting PTEN is miR-21, which has been investigated in numerous inflammatory diseases and malignancies [60,61,62]. In sepsis-related renal injury, the introduction of miR-21 significantly reduced the renal injury score [63]. Interestingly, miR-21 also contributes to sepsis protection induced by short periods of ischemia/reperfusion in femoral arteries. Specifically, sepsis-induced kidney damage was not prevented in miR-21 knockout animals [64]. In contrast to these studies, Wei et al. found that miR-21 can contribute to sepsis-related kidney damage by targeting cyclin-dependent kinase 6 (CDK6) [65]. Therefore, these studies demonstrate different mechanisms induced by a particular miRNA due to its involvement in different signaling pathways. Furthermore, different disease models can also alter miRNA functionality. In UUO mouse models, miR-21 encapsulated in the exosomes enhanced the fibrotic processes occurring in the kidneys [66].

Apart from the molecules described above, there is a growing number of investigated molecules regulating inflammation in sepsis-associated AKI. For instance, the expression of miR-128-3p is elevated in in vitro and in vivo sepsis models. By targeting neuropilin 1 (NRP1), miR-128-3p was found to stimulate the expression of pro-inflammatory mediators such as IL-6, TNF-α, IL-1β, and NF-κB. Importantly, introducing the inhibitor of miR-128-3p suppressed renal damage by reducing inflammation and improving kidney histology [67]. According to the literature, miR-128-3p can exert both pro- and anti-inflammatory features depending on the investigated cells and disease models [68,69,70]. However, in another study examining the involvement of miR-128-3p in sepsis, the authors observed that treatment of HK-2 cells with LPS downregulated the expression of miR-128-3p. Overexpression of this miRNA could suppress the pro-inflammatory response induced by LPS. Mechanistically, miR-128-3p regulated the expression of transforming growth factor beta receptor II (TGFBR2) [71]. Thus, the cellular context needs to be investigated to understand which molecule is being preferentially targeted by a particular miRNA.

Another group of miRNAs that regulate renal function under septic conditions are molecules regulating the expression of B-cell lymphoma-2 (BCL-2). BCL-2 is a group of proteins that regulate cell death and survival, named after the first member to be discovered, which has anti-apoptotic features [72]. miR-16-5p exacerbates the LPS-induced inflammatory responses in HK-2 cells. By targeting BCL-2, it could regulate apoptosis [73]. Similar results have been obtained regarding miR543 [74]. Other miRNA molecules that regulate inflammatory pathways in the development of AKI include miR-34b-5p [75], miR-181a-2-3p [76], miR-150-5p [77], and miR-20a-3p [78], among others. The abundance of such studies highlights the impressive number of regulatory mechanisms regulating apoptosis and inflammatory processes in AKI (Table 1).

### 2.3. miRNAs in AKI Induced by Nephrotoxic Agents

In the previous section, we focused on AKI that develops under septic conditions. However, kidney damage due to nephrotoxic agents is another significant challenge with complex mechanisms involved in its pathophysiology. For example, anticancer cytostatic drugs are known for their nephrotoxicity. Cisplatin is a frequently used chemotherapeutic agent used in the treatment of various neoplasms. Recent studies have demonstrated that alterations in the miRNA profile are involved in the pathogenesis of cisplatin-induced AKI. 

In an analysis by Wu and colleagues, treatment of HK-2 cells with cisplatin significantly altered the expression of 47 miRNAs [79]. Induction of cisplatin-related AKI (cis-AKI) is associated with increased expression of miR-483-5p, overexpression of which further deteriorates kidney function. Mechanistically, the molecule dysregulates apoptosis and autophagy of tubular epithelial cells by targeting GPX3, a member of the glutathione peroxidase family [80]. GPX3 was confirmed to be important in renal health in another study by Wu et al., who demonstrated that overexpression of GPX3 reduced the damage induced by ischemia-reperfusion [81]. Intriguingly, another member of the family, GPX4, was also found to mediate cisplatin-dependent AKI. Specifically, in in vitro and in vivo experiments, cis-AKI was associated with the elevated expression of miR-214-3p, a molecule that targeted GPX4. Suppression of miR-214-3p inhibited ferroptosis, a type of programmed cell death implicated in the pathogenesis of AKI [82,83]. In addition, cisplatin enhances another type of programmed cell death that is associated with inflammation: pyroptosis. Zhu and collaborators showed that cisplatin could enhance the pyroptosis of renal cells and stimulated these cells to secrete exosomes that prompted surrounding cells to initiate the programmed cell death process. Cellular death was suppressed by miR-122, which indicates the need for further research involving this molecule and renal damage [84].

DNA damage is also involved in the pathogenesis of cisplatin-induced renal damage. The metabolism of cisplatin involves modifications that allow it to bind to DNA, which then activates the DNA damage response signaling (DDR) pathway. Chronic activation of this pathway eventually leads to cell dysfunction or death [85]. A recent study demonstrated that cisplatin-induced DDR signaling could involve IL-22 signaling [86]. Intriguingly, repeated injections of cisplatin in mice causes DNA damage that leads to the state of CKD [87]. miRNAs were also suggested to mediate DNA damage processes. Yin and colleagues found that miR-155 knockout was associated with reduced accumulation of the DNA damage marker γH2AX. Moreover, the inhibition of miR-155 expression protected against apoptosis stimulated by the chemotherapeutic agent [88].

**Figure 2:**
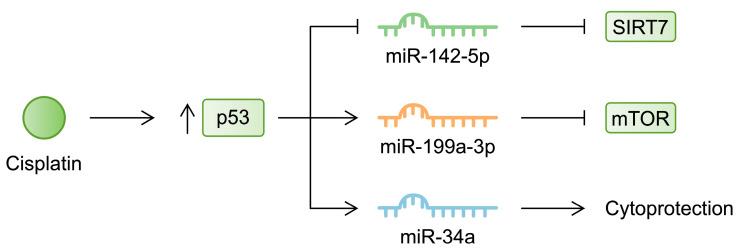
Cisplatin stimulates p53 activity, which then affects the expression of several miRNA molecules.

Similarly to sepsis-induced AKI, the nephrotoxic activity of cisplatin alters signaling pathways, and as previously mentioned, miRNAs are major regulators of signaling cascades. For instance, cisplatin was found to induce apoptosis and fibrosis in proximal tubular cells, and inhibition of p53 reduced these responses [89]. According to the previously mentioned study by Wu et al., the p53 pathway was among the most upregulated pathways in HK-2 cells treated with cisplatin [79]. P53 is a tumor suppressor that regulates the cell cycle and apoptosis by stimulating pro-apoptotic molecules such as PUMA. Cisplatin stimulates the expression of PUMA in renal cells, thus demonstrating its pro-apoptotic effects [90]. The p53 pathway is affected by miRNA activity, which ultimately modulates cisplatin-induced alterations. Specifically, overexpression of miR-142-5p suppressed the apoptosis of HK-2 cells that was enhanced by cisplatin. Inhibition of p53, which was activated by cisplatin, increased the expression of miR-142-5p. Mechanistically, miR-142-5p targets sirtuin 7 (SIRT7), a member of the SIRT family that has previously been suggested to be a therapeutic target in cis-AKI [91,92]. In another study, the expression of miR-199a-3p was found to be p53-dependent. Treatment of HK-2 cells with cisplatin was associated with increased expression of this miRNA, which was diminished in the presence of a p53 inhibitor. In turn, miR-199a-3p targeted and inhibited mTOR, a major regulator of cell survival, apoptosis, and autophagy [93]. In a recent review by Wang and collaborators, the authors discussed the involvement of mTOR in the pathogenesis of cis-AKI. The authors highlighted the complex role of mTOR in regulating the progression of the disease, but suggested that it plays a protective role [94]. Studies investigating the role of particular signaling cascades frequently demonstrate conflicting results, which shows the complex regulatory mechanisms that may depend on the cellular context. In the case of p53, Bhatt et al. found that cisplatin enhanced the expression of miR-34a via a p53-dependent mechanism. However, in contrast to miR-199a-3p, this molecule was suggested to play a protective role, as its inhibition further increased cell damage [95] (Figure 2). In addition, miRNAs can indirectly affect p53. According to Qin et al., treatment with cisplatin upregulated miR-449 in rat proximal tubular cells. Inhibiting the expression of this molecule stimulated the viability of renal cells. The molecule mediated the expression of SIRT1, which was associated with altered p53 acetylation status [96]. Consequently, the transcriptional properties of p53 changed, as described in more detail in a comprehensive review by Nagasaka and colleagues [97]. Moreover, other studies described the involvement of miRNAs in regulating other signaling cascades. For instance, cisplatin downregulates miR-30e-5p in mouse models. In an in vitro experiment, transfection of miR-30e-5p improved the viability of cisplatin-treated renal cells. Mechanistically, the molecule regulated the activity of the adenosine 5‘-monophosphate-activated protein kinase (AMPK) pathway [98]. Table 2 summarizes the expression profile and mechanisms linking miRNAs with cis-AKI. The studies discussed highlight the importance of miRNAs in renal injuries induced by nephrotoxic agents. Importantly, regeneration from cisplatin also involves miRNA modification. In a study by Candido de Almeida, the authors proved that administration of mesenchymal stem cells (MSCs) and MSC-derived microvesicles to cisplatin-pretreated mice stimulates the regeneration of damaged kidneys. Mechanistically, the authors showed that treatment with MSCs upregulated and downregulated 50 and 11 miRNAs, respectively. Furthermore, this treatment strategy restored physiological levels of proteins involved in the biogenesis of miRNAs [99]. Thus, this study proves the powerful impact of miRNAs in kidney functionality. Specifically, both damaging molecules, as well as regenerative therapies, alter miRNA expression profiles, which changes the renal parameters. 

### 2.4. Ischemia-Reperfusion AKI

It is estimated that up to 25% of cardiac output is delivered to the kidneys. However, as the oxygen tension is low, alterations in blood delivery may create an imbalance between nutrient and oxygen availability, resulting in organ injury. In the case of mild injuries, repair processes can restore organ function. In severe damage, patients may develop CKD, which leads to progressive organ dysfunction. Ischemia-reperfusion injury (IRI) is associated with the activation of the HIF and NF-κB pathways, as well as inflammatory responses [100]. Recent studies have demonstrated that the expression of miRNAs is also dysregulated following IRI, which could contribute to the progression of AKI.

The expression of miR-182 is upregulated in the renal tissue of rats exposed to IRI. This molecule was found to enhance apoptosis in renal tubular epithelial cells and to target the forkhead box O3 (FoxO3) transcription factor [101], which plays a protective role in hypoxic renal injury by regulating autophagy and oxidative stress responses [102]. FoxO3 is a transcription factor involved in major cellular processes, such as proliferation and metabolism. However, it is difficult to precisely demonstrate its effects on the pathogenesis of AKI, as its activity depends on cellular location and modification. In the context of hypoxia, FoxO3 has also been suggested to induce pro-apoptotic effects, which can be compensated for by the activity of deacetylase SIRT1 [103], thus suggesting the beneficial effects induced by SIRT1. In line with these findings, renal IRI enhances the expression of miR-132-3p, which targets and downregulates SIRT1 [104]. 

In the paragraph discussing sepsis-associated AKI, we mentioned the involvement of miR-21 in the pathogenesis of renal damage. Intriguingly, studies have also examined the role of this molecule in renal IRI. Specifically, the molecule is negatively associated with the maturation of dendritic cells (DCs). In contrast to immature DCs, mature cells stimulate inflammatory responses. Suppressing the expression of miR-21 in kidneys exposed to IRI was found to exacerbate the inflammatory response by increasing the expression of NF-κB and pro-inflammatory cytokines [105]. Moreover, miR-21 mimics inhibit DC maturation and thus reduce the expression of TNF-α and IL-6. Interestingly, injection of miR-21-overexpressing DCs into mice after IRI suppressed pro-inflammatory cytokines, along with the expression of NF-κB. Importantly, the transfer significantly reduced the serum creatinine level after 24 h of IRI [106]. Additionally, miR-21 was found to mediate the renoprotective properties of ghrelin [107], enhance proliferation, and suppress the apoptosis of renal cells in gentamicin-induced kidney injury in fish models [108]. In addition, miR-211 [109], miR-192-5p [110], miR-187 [111], and miR-194 [112] were also investigated in the context of IRI/hypoxia and reoxygenation (Table 3). Recently, Chen et al. demonstrated that miR-155-3p contributes to the previously mentioned cell death, pyroptosis. Intriguingly, the authors found that miR-155-3p is transferred into adjacent cells through gap junctions formed by connexin 32, inhibition of which could suppress pyroptosis [113]. Additionally, researchers suggest the important involvement of PTEN in regulating IRI-based kidney injury. miR-486-5p, a molecule that targets PTEN, induced protective mechanisms in IRI mice [114]. However, as with other miRNAs, miR-486-5p has several targets and its influence on kidney functionality may depend on other pathways. Vinas and collaborators showed that apart from downregulating PTEN, miR-486-5p suppresses the expression of TNF and apoptotic genes [115]. The activity of miR-17-5p was also found to induce protective effects on kidney function in the IRI mice model. This molecule also targets the expression of PTEN [116]. However, as pharmacological inhibition of PTEN was associated with more pronounced kidney damage in the IRI context [117], the protective role of miRNAs could depend on their broad regulatory network, which also involves PTEN. Regarding miR-17, the molecule belongs to the cluster known as miR-17~92, which contains several miRNAs that regulate major cellular processes and are frequently dysregulated in a number of pathological conditions [118]. The cluster has important proangiogenic properties which are suggested to induce positive effects under ischemic conditions. Accordingly, the loss of function of the cluster in renal endothelial cells was associated with enhanced renal damage after IRI. By contrast, administration of miR-18a and miR-18b mimics (members of the miR-17~92 cluster) stimulated renal recovery [119].

As previously mentioned, ischemic conditions enhance the expression of HIF-1, which regulates the important processes of angiogenesis, survival, and metabolic adaptation [120]. Researchers demonstrated that HIF-1 induces protective effects by regulating the expression of miRNAs. According to Wei et al., HIF-1 upregulates miR-668, a molecule that has antiapoptotic properties. Mechanistically, this miRNA prevented mitochondrial fragmentation by binding to the mitochondrial protein 18 kDa (MTP18) [121]. Similarly, HIF-1 enhances the expression of miR-489, which targets PARP1 and suppresses apoptosis [122].

## 3. microRNA in Clinical Studies and Their Role as Diagnostic Biomarkers

In previous sections, we have discussed the involvement of miRNA molecules in various pathways associated with the pathogenesis of AKI. It is considered that abnormal expression of these molecules dysregulates signaling pathways, which eventually stimulates pathological mechanisms in the kidney. From the clinical perspective, altered levels of miRNAs could be monitored to detect the developing AKI. Under septic conditions, 27 down- and 13 upregulated molecules were detected in patients who developed AKI [30].

Indeed, the potential role of miRNAs as diagnostic biomarkers has been investigated. For instance, a recent study by Aomatsu et al. suggested that monitoring the serum expression of miR-5100 could distinguish patients with AKI from healthy volunteers. The authors observed a reduced expression of this miRNA in the serum of patients. The receiver operating curve (ROC) analysis highlighted high diagnostic potential with the area under curve (AUC) set at 0.96 [123]. Another approach to the use of miRNAs in the diagnosis of AKI could involve the identification of hub genes significantly associated with the disease. Subsequently, a regulatory set of miRNA molecules could be investigated to create a diagnostic panel. Sun and colleagues performed a similar study and identified 15 hub genes for critically ill patients with AKI. The authors found that miR-6884-5p, miR-532-5p, miR-218-5p, miR-485-5p, and miR-181c-5p represent enriched molecules associated with the identified hub genes [124]. Importantly, these novel molecular biomarkers could be used to detect AKI in patients with different background conditions, thus creating an opportunity for personalized diagnosis panels. Recently, a relationship between the altered expression of miR-22-3p and the occurrence of AKI was observed. We have previously mentioned that downregulated miR-22-3p has been associated with worse kidney functionality in AKI in preclinical models. In septic patients, the occurrence of AKI and the increased severity of the condition reduced the expression of miR-22-3p in serum and urinary samples. The diagnostic potential of using the AUC was 0.851 for serum and 0.911 for urinary miRNA expression, suggesting its promising diagnostic accuracy. Interestingly, Zhang et al. showed that lower expression of miR-22-3p is also associated with worse survival rates [125]. In contrast-induced AKI, the AUC value of circulating miR-188-5p was 0.725 [126]. Furthermore, another condition associated with impaired renal function is acute decompensated heart failure (ADHF) [127]. miR-652-3p and miR-423-5p represent miRNAs recently suggested to have a promising diagnostic potential in diagnosing AKI in patients with ADHF [128,129]. Additionally, miRNAs could potentially serve as diagnostic biomarkers of AKI in transplant recipients, as they are suggested to be involved in the expression and regulation of hub genes associated with renal impairment [130]. Furthermore, miR-21 has been investigated in the diagnostic context. Gaede et al. demonstrated that monitoring of serum miR-21 could predict the occurrence of AKI in patients after major cardiac surgery [131]. Thus, current evidence demonstrates the broad and promising uses of miRNAs as clinical biomarkers in patients with AKI. 

## 4. miRNA-Based Therapeutics

This review, together with numerous other papers, discusses the involvement of the dysregulated expression of miRNAs in the pathogenesis of disease. These pieces of knowledge suggest novel therapeutic targets that could be used in treatments. However, knowledge about the use of miRNA-based therapeutics is still limited; it mainly comes from in vivo animal experiments, and there are very few examples of clinical research articles. For instance, in a study by Deng et al., the use of miR-206 agomir delayed preterm births in mice [132]. In cases of miRNAs contributing to the progression of disorders, these molecules can be targeted by oligonucleotides to reduce their expression. Miravirsen is an miR-122-targeting oligonucleotide that is being investigated in patients with chronic HCV infection, a condition in which miR-122 plays a role in pathogenesis [133]. 

Unfortunately, systemic administration of miRNA-based therapeutics is associated with certain limitations. These molecules can undergo degradation by RNases or cellular endocytic compartments [134]. To prevent degradation, therapeutics can be modified or encapsulated. This approach has been examined in oncological treatments implementing miRNAs. For example, MRX34 is a liposomal miR-34a mimic, which has been investigated as a potential therapeutic in solid tumors. However, a phase 1 clinical trial was terminated due to adverse events [135], which demonstrates another limitation of miRNA-based drugs that needs to be eliminated. Currently, a significant number of studies are needed to try to form an effective and safe delivery method for miRNA therapeutics [136,137]. 

## 5. Conclusions

To conclude, numerous miRNAs have been found to contribute to the pathogenesis of AKI. Various renal injury models, such as sepsis and drug-induced nephrotoxicity, as well as IRI, alter the expression of miRNA, thus changing the gene expression profile. The dysregulated profile of miRNAs is associated with abnormal inflammatory responses, fibrosis, and renal cell apoptosis, as well as abnormal signaling pathways, leading to abnormalities in cell viability and autophagy. Understanding these regulatory mechanisms could lead to the introduction of novel treatment methods that perhaps will induce renal regeneration more rapidly and prevent progression to CKD. Moreover, by accumulating knowledge regarding the expression of miRNAs in patients with AKI, recent studies have started evaluating their diagnostic potential. As conditions associated with renal impairment are frequently severe and involve multi-organ damage, such as sepsis, the use of miRNA biomarkers could help to precisely identify damaged organs or pathological conditions requiring interventions. 

## Figures and Tables

**Table 1:** Summary of microRNA molecules that regulate mechanisms associated with acute kidney injury under septic conditions.

MicroRNA	Mechanism Associated with Acute Kidney Injury	References
miR-23a-3p	miR-23a-3p negatively regulates FKBP5, thus suppressing the activity of the NF-κB and reducing pro-inflammatory response.	[31]
miR-23a-3p	miR-23a-3p suppresses kidney injury by targeting Wnt5, a member of the Wnt/β-catenin signaling pathway.	[33]
miR-106a	miR-106a further stimulates kidney cell injury by targeting the anti-angiogenic thrombospondin 2.	[42]
miR-106a-5p	miR-106a-5p targets HMGB1, which is associated with podocyte injury and the expression of which is elevated in mouse models of AKI.	[43,44]
miR-22	miR-22 suppresses septic AKI due to inhibiting the HMGB1/TLR4/NF-κB signaling pathway.	[45]
miR-26a-5p	miR-26a-5p induced protective mechanisms in septic models of AKI by targeting IL-6.	[51]
miR-22-3p	miR-22-3p suppresses inflammatory responses in animal sepsis AKI models and LPS-stimulated HK-2 cells by targeting PTEN.	[57]
miR-93	miR-93 targets PTEN and activates the signaling of the AKT/mTOR pathway, which is associated with improved viability in LPS-pretreated renal cells.	[58]
miR-214	By targeting PTEN, miR-214 improved renal parameters, suppressed inflammation, and improves renal histopathology in septic mice.	[59]
miR-21	miR-21 can suppress renal injury induced by sepsis by targeting PTEN.	[63]
miR-21	miR-21 contributes to the kidney damage induced by LPS by targeting CDK6.	[65]
miR-128-3p	miR-128-3p contributes to sepsis-associated kidney damage by targeting NRP1 and enhancing inflammatory response.	[67]
miR-16-5p	By targeting BCL-2, miR-16-5p regulates apoptosis and further stimulates LPS-induced inflammation.	[73]
miR-543	Downregulating miR-543, which targets BCL-2, reduces inflammatory response and apoptosis.	[74]

**Table 2:** Summary of expression profile of selected microRNAs and mechanisms associated with cisplatin-induced AKI.

MicroRNA	Expression in Cisplatin-Induced AKI (In Vitro or In Vivo)	Mechanism	References
miR-483-5p	Increased	miR-483-5p disrupted apoptosis and autophagy in renal cells through targeting GPX3, a member of the glutathione peroxidase family.	[80]
miR-214-3p	Increased	miR-214-3p regulates ferroptosis by targeting GPX4.	[82]
miR-155	Increased	Targeting miR-155 suppresses cisplatin-induced DNA damage.	[88]
miR-142-5p	Decreased	Suppression of p53 enhances the expression of miR-142-5p, which inhibits apoptosis induced by cisplatin.	[91]
miR-199a-3p	Increased	Cisplatin-induced expression of miR-199a-3p was p53-dependent, and the molecule regulated the mTOR gene.	[93]
miR-34a	Increased	miR-34a has cytoprotective features as its inhibition further enhanced cisplatin-induced cell damage.	[95]
miR-449	Increased	Suppression of miR-449 enhances renal cell viability.	[96]
miR-30e-5p	Decreased	Overexpression of miR-30e-5p improves cell viability in cisplatin-treated renal cells. The molecule regulates the AMPK pathway.	[98]

**Table 3:** Summary of selected microRNAs with known altered expression profiles in IRI-associated AKI, which contributes to the pathogenesis of renal injury.

MicroRNA	Impact of Ischemia-Reperfusion Injury on Expression in Renal Tissue/Cells	Mechanism Linking to Acute Kidney Injury	References
miR-182	Increased	miR-182 enhances apoptosis of tubular epithelial cells through targeting FoxO3.	[101]
miR-132-3p	Increased	miR-132-3p targets SIRT1 and further deteriorates oxidative balance in ischemia.	[104]
miR-21	Increased	miR-21 plays a protective role in IRI-associated AKI by suppressing dendritic cell maturation, thus limiting the inflammatory responses.	[105]
miR-211	Decreased	Through inhibiting TGF-β/SMAD signaling, miR-211 improves cell viability.	[109]
miR-192-5p	Increased	Suppression of miR-192-5p improved viability of renal cells under hypoxia/reoxygenation conditions. The molecule targets FTO.	[110]
miR-187	Decreased	Increasing the expression of miR-187 reduces podocyte damage.	[111]
miR-194	Decreased	By targeting Rheb, miR-194 reduces inflammation and oxidative stress in renal cells stimulated by hypoxia and reoxygenation.	[112]

## Data Availability

Not applicable.
